# Association of a probiotic to a *Helicobacter pylori* eradication regimen does not increase efficacy or decreases the adverse effects of the treatment: a prospective, randomized, double-blind, placebo-controlled study

**DOI:** 10.1186/1471-230X-13-56

**Published:** 2013-03-26

**Authors:** Tomás Navarro-Rodriguez, Fernando Marcuz Silva, Ricardo Correa Barbuti, Rejane Mattar, Joaquim Prado Moraes-Filho, Maricê Nogueira de Oliveira, Cristina S Bogsan, Décio Chinzon, Jaime Natan Eisig

**Affiliations:** 1 Serviço de Gastroenterologia Clínica do Hospital das Clínicas da Faculdade de Medicina da Universidade de São Paulo, Av. Dr. Enéas de Carvalho Aguiar, 255 – Cerqueira Cezar, São Paulo, SP, Brasil

**Keywords:** *Helicobacter pylori* eradication, Probiotic, Tetracycline, Furazolidone, Peptic ulcer, Functional dyspepsia, Treatment efficacy, Adverse effects

## Abstract

**Background:**

The treatment for the eradication of *Helicobacter pylori* (*H. pylori*) is complex; full effectiveness is rarely achieved and it has many adverse effects. In developing countries, increased resistance to antibiotics and its cost make eradication more difficult. Probiotics can reduce adverse effects and improve the infection treatment efficacy.

If the first-line therapy fails a second-line treatment using tetracycline, furazolidone and proton-pump inhibitors has been effective and low cost in Brazil; however it implies in a lot of adverse effects. The aim of this study was to minimize the adverse effects and increase the eradication rate applying the association of a probiotic compound to second-line therapy regimen.

**Methods:**

Patients with peptic ulcer or functional dyspepsia infected by *H. pylori* were randomized to treatment with the furazolidone, tetracycline and lansoprazole regimen, twice a day for 7 days. In a double-blind study, patients received placebo or a probiotic compound (*Lactobacillus acidophilus, Lactobacillus rhamnosus, Bifidobacterium bifidum* and *Streptococcus faecium*) in capsules, twice a day for 30 days. A symptom questionnaire was administered in day zero, after completion of antibiotic therapy, after the probiotic use and eight weeks after the end of the treatment. Upper digestive endoscopy, histological assessment, rapid urease test and breath test were performed before and eight weeks after eradication treatment.

**Results:**

One hundred and seven patients were enrolled: 21 men with active probiotic and 19 with placebo plus 34 women with active probiotic and 33 with placebo comprising a total of 55 patients with active probiotic and 52 with placebo. Fifty-one patients had peptic ulcer and 56 were diagnosed as functional dyspepsia. The per-protocol eradication rate with active probiotic was 89.8% and with placebo, 85.1% (p = 0.49); per intention to treat, 81.8% and 79.6%, respectively (p = 0.53). The rate of adverse effects at 7 days with the active probiotic was 59.3% and 71.2% with placebo (p = 0.20). At 30 days, it was 44.9% and 60.4%, respectively (p = 0.08).

**Conclusions:**

The use of this probiotic compound compared to placebo in the proposed regimen in Brazilian patients with peptic ulcer or functional dyspepsia showed no significant difference in efficacy or adverse effects.

**Trial registration:**

Current Controlled Trials ISRCTN04714018

## Background

The *Helicobacter pylori* (*H. pylori*) eradication is a very useful tool in preventing the recurrence of peptic ulcer, in the prevention of gastric cancer and in the treatment of Malt lymphoma and gastritis [1-4]. However, the regimens used to *H. pylori* eradication are complex and require the combination of at least two antibiotics with acid suppressors that must be administered for several days [5,6]. Therefore they can lead to many adverse effects [7], most of them related to the medication but also alterations in intestinal bacterial flora due to antibiotic treatment can occur [8,9]. Most patients have trouble to adhere to treatment and to purchase the drugs that makes the eradication even more difficult.

Brazil is a densely inhabited developing country with a rate of *H. pylori* infection ranging from 58 to 80% of the adult population [10-17] and most of the infected population has low socioeconomic level. Due to specific characteristics (antibiotic prophylaxis for surgery and treatment of parasitic infections and or sexually transmitted diseases) the primary resistance to antibiotics used in the eradication of *H. pylori* is high (e.g., nitroimidazole derivatives) [18-23]. Thus, *H. pylori* eradication in Brazil should focus on the use of regimens with shorter duration and lower cost using highly effective antibiotics. The treatment of choice is the combination of proton pump inhibitor with clarithromycin and amoxicillin which has an efficacy of 85% [24].

The regimen that includes tetracycline, furazolidone and omeprazole for 7 days has been used as a second-line treatment, with 75% efficacy [25]; on the other hand furazolidone results in many adverse effects.

Adverse effects are a common cause of lack of adherence to treatment leading to treatment failure in eradicating *H. pylori*[6,26]. Treatment failure results in a higher risk of secondary resistance to antibiotics [27] and will require the use of new and usually less effective ones, with longer duration, higher cost and complex regimens [28].

Some adverse effects of treatment are intestinal alterations secondary to changes in the microbiota due to antibiotic use [29,30]. Probiotics would be an excellent tool in controlling bacterial overgrowth and reduce these effects [31-34]. There is also evidence that probiotics may also inhibit the growth of *H. pylori*, stimulate an immunological response and reduce the inflammatory effects of infection by bacteria [35-37] increasing the rate of *H. pylori* eradication [38-40].

This study aimed to detect the rate of eradication efficacy and of side effects in *H. pylori* treatment with the combination of a probiotic compound and the regimen lansoprazole, furazolidone and tetracycline in a prospective, randomized, double-blind, placebo-controlled study.

## Methods

### Patients

Developed in 2007 the treatment design had no previous data in literature for the proposed regimen: association of 7 days of treatment to *H. pylori* eradication added to 30 days of probiotic, both initiating at the same time. The sample calculation was based on a previous study [25] with a similar regimen, in which 20 mg of omeprazole was once a day intake, with tetracycline 500 mg and furazolidone 200 mg three times a day, for 7 days. By Fisher's exact test, with unilateral hypothesis, expecting an increase in the eradication rate of 70 to 90% with probiotic use, for a power of 0.80 and a significance level of 0.05, the sample size was 56 patients for each group, active or placebo. For a 34 to 15% reduction in the rate of adverse events with probiotic use, for a power of 0.80 and a significance level of 0.05, the sample size was 60 patients for each group.

Patients infected with *H. pylori* with a previous diagnosis of peptic ulcer or functional dyspepsia were invited to participate in the study.

Inclusion criteria were: more than 18 years old, no previous treatment for the infection, not having a chronic decompensated disease, no use of anti-inflammatory or antibiotic drugs within 4 weeks prior to enrollment and sign of informed consent.

Exclusion criteria were: patients who were pregnant or breastfeeding, patients over 80 years of age or with a history of gastrointestinal surgery and patients with erosive esophagitis or users of low-dose aspirin. Patients with difficulty to understand the treatment or to report disease symptoms and adverse effects were also excluded.

All patients were followed at the Gastroenterology outpatient clinic of this Hospital and had been previously diagnosed by upper digestive endoscopy. The study was approved by the ethics and research committee of the institution: Ethics Committee for Analysis of Research Projects - CAPPesq – of the Clinical Board of Clinics Hospital, Faculty of Medicine, University of São Paulo.

### Diagnoses

All patients were underwent carbon-13 labeled urea breath test and underwent upper digestive endoscopy. If a patient was taking proton-pump inhibitors or H_2_ blockers they discontinued it 10 days before the beginning of the study. The symptomatic use of aluminum hydroxide was allowed until the beginning of treatment. During endoscopy mucosal fragments were collected to perform histological assessment with HE and Giemsa staining and rapid urease test from the gastric antrum and gastric body. Eight weeks after treatment completion patients with peptic ulcer were submitted to the same examinations and those with functional dyspepsia underwent urea breath test for heal monitoring.

### Symptom questionnaire

**Figure 1:**
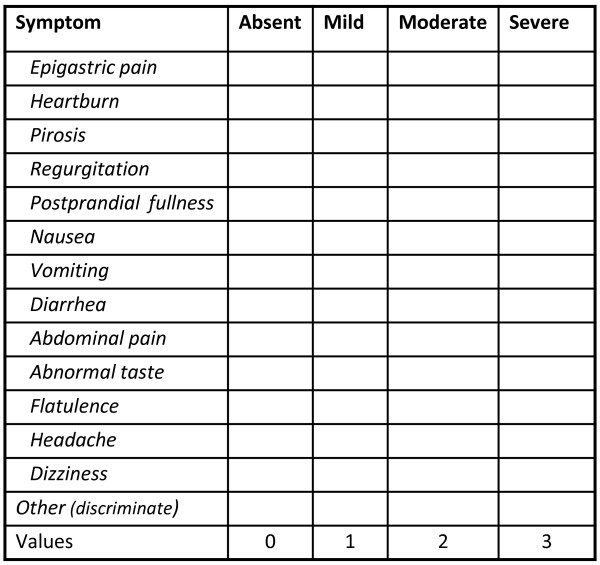
**Symptom Questionnaire. **Absent: without symptom; Mild: symptom doesn’t interfere with normal activity; Moderate: symptom interferes with normal activity < 50% of evaluated time; Severe: Symptom interferes with normal activity > 50% of evaluated time, or daily, or determines the treatment interruption.

Before treatment, on the seventh and thirtieth day of treatment and 60 days after its completion, all patients answered a questionnaire on dyspeptic symptoms and the most common symptoms related to possible adverse effects due to treatment (Figure 1). Each symptom was quantified as absent (zero), mild (1), moderate (2) and severe (3). The questionnaire allowed the inclusion of new symptoms (considered as adverse effects) in the assessments after drug administration. We determined the number and intensity of symptoms in all patient evaluations. Previous symptoms that increased in intensity during and after treatment were also considered as adverse effects.

### Treatment

The patients received tablets and capsules in adequate amount for 7 days of treatment, administered twice a day: 30 mg of lansoprazole, 500 mg of tetracycline and 200 mg of furazolidone. The probiotic compound consisting of *Lactobacillus acidophilus* (1.25 × 10^9^ CFUs)*, Lactobacillus rhamnosus* (1.25 × 10^9^ CFUs)*, Bifidobacterium bifidum* (1.25 × 10^9^ CFUs) and *Streptococcus faecium* (1.25 × 10^9^ CFUs) (Klaire Labs, Reno, NV, USA) was provided in a bottle with 60 capsules and instructions to be stored in the refrigerator and to be used regularly during 30 days (7 days of treatment plus 23 days after cessation of the antibiotic). The placebo probiotic consisting of capsules of acidified milk powder (skim milk biologically acidified by commercial yogurt culture) was also provided at the same amount and with the same instructions.

All patients were taught at the sight of medication by the researcher himself and encouraged to maintain full and regular use of medication considering the benefits of eradicating *H. pylori*. They were also asked to maintain complete abstinence of alcohol, to hamper smoked foods, chocolate, cheese and eggs and not to use antidepressants to avoid interaction with Monoamine Oxidase Inhibitor-like effects of furazolidone during the treatment.

### Randomization and study performance

Randomization was carried out using a list obtained by computer. Patients received their numbers in ascending order according to study enrollment. This number corresponded to the randomized regimen for use of medication with an active or placebo probiotic.

None of the patients knew about the randomization and investigators, blinded to the randomization, followed the treatment and performed all examinations independently. The need to disclose the randomization always resulted in the exclusion of the patient.

Drug use control was performed 7 and 30 days after the delivery of medication by counting the remaining tablets in the blisters and the number of probiotic capsules in the bottle. The use of at least 80% of the tablets was considered appropriate.

Patients with peptic ulcer were considered cured of infection when they had negative treatment control tests: rapid urease test, histological analysis and breath test, performed 60 days after the treatment. For patients with functional dyspepsia, they were considered cured when they had a negative urea breath test.

After treatment completion, up to control tests, patients with severe epigastric pain or heartburn symptoms were allowed to use aluminum hydroxide pills symptomatically. The use of any amount of the antacid agent characterized the intensity of the symptom as severe.

### Statistical analysis

The eradication rates were analyzed per intention to treat and per protocol, with 95% confidence interval (*P*<0.05).

The Chi-square test method with Pearson’s correction factor was used to compare variables and a p value < 0.05 was considered statistically significant. The homogeneity of the groups was evaluated using the nonparametric Chi-square test.

The distribution of patients in the active probiotic and placebo groups, age and gender, both for ulcer disease and for functional dyspepsia, was also evaluated by the Mann–Whitney test.

The analysis of adverse effect incidence was determined by Pearson Chi-square test and the intensity of adverse events at the 7 and 30 day control visits were evaluated by Mann–Whitney test, using the score obtained from symptom questionnaire.

The frequencies of the variables, the percentage of tests and measurements were carried out using Statistical Package for the Social Sciences, 10.0 Version (SPSS Inc., Chicago, IL, EUA).

## Results

### Data of included population

**Figure 2:**
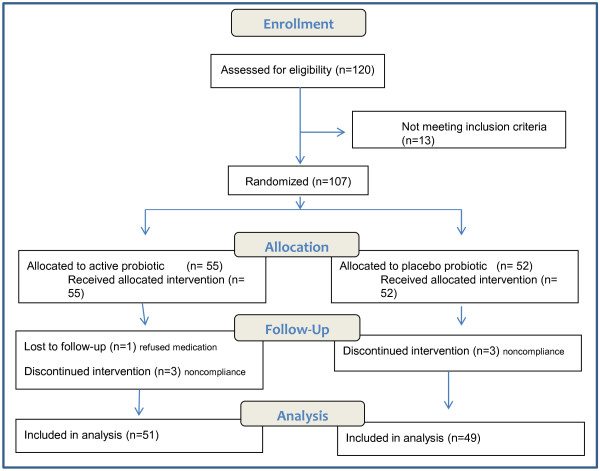
Probiotic Flow Diagram.

The flow diagram of patient enrollment in this study is shown in Figure 2.

One hundred and seven patients were included in the study, being 40 were men (37%) and 67 women (63%). The mean age was 47 years, with a median of 51 years, ranging from 21 to 74 years. Fifty-five patients (51%) were allocated in the active probiotic group and 52 (49%) in the placebo probiotic group. Fifty-one patients (48%) had peptic ulcer: duodenal 29 (57%), gastric 17 (33%) and 5 (10%) gastric and duodenal. Twenty-seven peptic ulcer patients were in the active probiotic group and 24 in the placebo group. Fifty-six patients (52%) had functional dyspepsia, with 28 in the active probiotic group and 28 in the placebo group (Table 1).

**Table 1:** Patient allocation in groups of active and placebo probiotic agent

		**Active**	**Placebo**	**p**
N		55	**52**	
Gender	male	21	19	
	female	34	33	0.86
Disease	peptic ulcer*	27	24	
	dyspepsia	28	28	0.76
Mean age (years)		50.4	48.4	0.78

Pearson Chi-Square.

*29 duodenal 17 gastric 5 gastric + duodenal.

### Follow up

One patient in the active probiotic group refused the medication regimen for eradication.

Six patients did not undergo eradication control: 3 due to inappropriate use of the eradication regimen (they were concerned with the adverse effects) and three abandoned the study after the eradication regimen use. They were equally divided in the two groups.

Four patients used the eradication regimen and came for adverse effect control after seven days but then discontinued probiotic use although they returned for eradication control. Three were in the placebo probiotic group and one in the active probiotic group.

### Eradication rates

The overall per protocol eradication rate was 87.5% and per intention to treat, 79.4%. There was no statistical difference in eradication rate between those who used active probiotic and placebo, both per protocol (89.8% × 85.1%) and per intention to treat (81.8% × 76.9%) Table 2.

**Table 2:** Eradication rates in groups of active and placebo probiotic

**Probiotic**	**Active (CI 95%)**	**Placebo (CI 95%)**	**p**
Per protocol	89.8% (81-99%)	85.1% (75-96%)	p = 0.49
Intention to treat	81.8% (71-92%)	76.9% (65-89%)	p = 0.53

Pearson Chi-Square.

There was not statistical difference regarding the efficacy of eradication per-protocol among patients with peptic ulcer (91.1%) and those with dyspepsia (84.3%), with p = 0.31. Moreover per-intention to treat also did not present statistical difference (ulcer = 80.4% dyspeptic = 78.6% p = 0.81).

Although per-protocol efficacy of eradication among ulcer patients who used active probiotic was higher than those who used placebo probiotic, the rate was no statistically significant (p = 0.23). Findings were similar for patients with functional dyspepsia (Table 3).

**Table 3:** Eradication rates per protocol in peptic ulcer disease and dyspepsia in groups of active and placebo probiotic

	**Peptic ulcer (CI 95%)**	**Dyspepsia (CI 95%)**
Active probiotic	95.8% (87-100%)	84.0% (74-100%)
Placebo probiotic	85.7% (69-100%)	84.6% (70-99%)
Pearson Chi-Square	p = 0.23	p = 0.95

### Adverse effects

At the 7-day visit 69 of 106 (65%) of patients reported side effects and at the 30-day visit, 50 of 97 patients were still reporting them (52%). Although there were differences regarding the incidence of adverse effects among patients who used active probiotic and the placebo group they did not reach statistical significance (Table 4).

**Table 4:** Incidence of adverse effects in groups of active and placebo probiotic at 7 and 30-day visits

**Visit**	**Active probiotic**	**Placebo probiotic**	**p**
7 day	32/54 (59.3%)	37/52 (71.2%)	0.20
30 day	21/49 (44.9%)	29/48 (60.4%)	0.08

Pearson Chi-Square.

The mean intensity of the adverse effects determined at 7 and 30 days was not different between groups of active probiotic and placebo (Table 5).

**Table 5:** Intensity of adverse effects in groups of active and placebo probiotic at 7 and 30 day visits

**Visits**	**Active probiotic**	**Placebo probiotic**	**p**
7 day mean/sum rank	49/2,642	58/3,028	0.11
30 day mean/sum rank	48/2,329	51/2,423	0.58

Mann–Whitney Test.

## Discussion

The triple treatment regimen used in this study favored the short-term therapy (7 days), convenient dosage (twice a day) and simplicity (3 units) aimed at low cost, easy understanding and greater adherence with antibiotics which in our country show low bacterial resistance. These characteristics make treatment adherence depend more on the adverse effects that treatment complexity when compared to the classic regimen including omeprazole, clarithromycin and amoxicillin [24].

The regimen used in this study differed from a previously tested treatment [25] with furazolidone and tetracycline administered 3 × day and the proton-pump inhibitor once a day; in this regimen the drugs were equally administered twice a day for 7 days. In the first regimen the eradication effectiveness per-protocol was 75%. In this study, the per-protocol eradication efficacy was higher: 87.5% (perhaps by greater simplicity of the regimen) and this rate was similar to that obtained in our country, with a regimen that included proton-pump inhibitor, clarithromycin and amoxicillin: 88.8% (per protocol), also a short-duration regimen, preferably used as first-line eradication of *H. pylori*[24]. The differences between the regimens, PPI + amoxicillin + clarithromycin twice a day for 7 days or PPI once a day + Tetracycline + Furazolidone 3 times a day for 7 days were the incidence of severe adverse effects that was much lower for clarithromycin with amoxicillin (3.7%) than with furazolidone with tetracycline (15%).

This study associated a probiotic compound to the antibiotic regimen targeting a lower rate of the adverse effects. The incidence of adverse effects were higher than expected probably because of the use of a standard symptom questionnaire. Unfortunately although there was a difference in incidence and severity of adverse effects between the active probiotic and placebo groups, no statistical significance was observed.

In the literature studies have observed increase of eradication rate and decrease of adverse effects in *H. pylori* eradication when a probiotic is associated with the treatment of the infection [39-43]. Ojetti and coworkers [44] used a single strain of lactobacillus with 1×10^8^ CFUs for 14 days also associated with a triple regimen of eradication (PPI + Levofloxacin + Amoxicillin) with 7 days in duration and obtained both increasing eradication and a reduction in adverse effects. Du and colleagues [45] also with a 7 days triple eradication regimen (PPI + Amoxicillin + Clarithomycin) also with 14 days treatment with a single strain of bacillus (3×10^7^ CFUs) in patient groups approximately equal to ours also obtained eradication increased and adverse effects decreased.

Others studies achieved a decrease of adverse effects without an increase of eradication rate [34,44,46,47]. Among them, one of Manfredi *at al.*[46] associated a compound of the four different probiotics and prebiotics for 10 days in a sequential treatment for eradication of *H. pylori* and observed a reduction of adverse effects, although no increase in eradication.

On the other hand some studies didn’t verified significant benefits in probiotic use [48-50]. Yoon [49] and coworkers joined a compound of 4 probiotics, for 4 weeks, to a treatment of 14 days second-line regimen for *H. pylori* eradication, with PPI + amoxicillin + moxifloxacin that did not increase eradication or reduce the adverse effects.

The different results are probably due to the different products used, their different concentrations, probiotic strain, dose and duration of use and also the strain of *H. pylori* in question, as suggested by Vitor [38] and Wilhelm [33].

Considering the fact that the use of a probiotic agent also adds more complexity to the process as it increases treatment duration, the benefit of attaining a lower incidence of adverse effects or higher eradication effectiveness with longer treatment duration is debatable, especially because the difference did not reach statistical significance, as also stated by Medeiros et al. [51].

However if probiotics can reduce the adverse effects of *H. pylori* eradication it could enable greater adherence to treatment and could increase the eradication rate by intention to treat.

Thus it is necessary to seek other probiotic combinations or other presentations or other dosages or other treatment duration to achieve these goals.

## Conclusions

The probiotic compound used in the present study (*Lactobacillus acidophilus*, *Lactobacillus rhamnosus*, *Bifidobacterium bifidum* and *Streptococcus Faecium*), administered for 30 days associated to the *H. pylori* eradication regimen: Lansoprazole 30 mg, Tetracycline 500 mg and Furazolidone 200 mg administered twice a day for 7 days did not show an increase in bacterial eradication effectiveness or decrease in adverse effects of *H. pylori* eradication treatment in Brazilian patients with peptic ulcer and functional dyspepsia.

## Competing interests

The authors declare they have no financial or non-financial competing interests.

## Authors’ contributions

All authors contributed to the design of the study. Acquisition of data and quality control: TNR, FMS, JNE, MNO, CSBB. Analysis and interpretation of data: TNR, FMS, JNE, MFoJP. Endoscopic examinations: RCB, DC Laboratory: RM. All authors have read and approved the final manuscript.

## Pre-publication history

The pre-publication history for this paper can be accessed here:

http://www.biomedcentral.com/1471-230X/13/56/prepub
